# TimeMeter assesses temporal gene expression similarity and identifies differentially progressing genes

**DOI:** 10.1093/nar/gkaa142

**Published:** 2020-03-03

**Authors:** Peng Jiang, Connie S Chamberlain, Ray Vanderby, James A Thomson, Ron Stewart

**Affiliations:** 1 Regenerative Biology Laboratory, Morgridge Institute for Research, Madison, WI 53707, USA; 2 Department of Orthopedics and Rehabilitation, University of Wisconsin, Madison, WI 53706, USA; 3 Department of Biomedical Engineering, University of Wisconsin, Madison, WI 53706, USA; 4 Department of Molecular, Cellular and Developmental Biology, University of California, Santa Barbara, CA 93106, USA

## Abstract

Comparative time series transcriptome analysis is a powerful tool to study development, evolution, aging, disease progression and cancer prognosis. We develop TimeMeter, a statistical method and tool to assess temporal gene expression similarity, and identify differentially progressing genes where one pattern is more temporally advanced than the other. We apply TimeMeter to several datasets, and show that TimeMeter is capable of characterizing complicated temporal gene expression associations. Interestingly, we find: (i) the measurement of differential progression provides a novel feature in addition to pattern similarity that can characterize early developmental divergence between two species; (ii) genes exhibiting similar temporal patterns between human and mouse during neural differentiation are under strong negative (purifying) selection during evolution; (iii) analysis of genes with similar temporal patterns in mouse digit regeneration and axolotl blastema differentiation reveals common gene groups for appendage regeneration with potential implications in regenerative medicine.

## INTRODUCTION

With the advance of high throughput methods, such as RNA-seq, the amount of time series gene expression data has grown rapidly, providing an unprecedented opportunity for comparative time series gene expression analysis. Although many studies are aimed at identifying differentially expressed genes (DEGs) (1,2) either between different time points along a time series (3–5) or between two conditions at the same time point (6,7), DEGs only signify snapshots of each individual time point comparison, and do not consider temporal dynamic change information. Hence one of the fundamental problems in time series gene expression data analysis is how to characterize time series gene expression dynamic changes, and capture temporal pattern associations. Although several methods have been developed to address this issue, they are mostly limited to either using correlation analysis by requiring gene expression values measured at the same time for both conditions (8), or aiming at identifying linear associations (e.g. similar patterns but patterns shifts overtime (‘time shift pattern’) (9–11)). Emerging evidence suggests that the majority of similar gene expression patterns are far more complicated than linearly associated patterns (12,13). For example, comparing human embryonic stem (ES) cells and mouse epiblast stem (EpiS) cells during neural differentiation, many genes in mouse EpiS cells exhibit nonlinearly faster dynamical changes when compared to human ES cells (13). The patterns can be even more complicated if they are a mixture of dynamical speed difference (e.g. faster or slower dynamical changes), time shifts, and dissimilar patterns over time.

Comparing time series gene expression data from different experiments requires computational methods that can handle sequences of different length and sampling density. Although studies using coarse-grained associations (simplifying dynamical changes to a few features, such as expression peaks (14)) can potentially solve some of these problems, and partially model complex temporal gene expression patterns, they may sacrifice temporal resolution. Hence, there is a pressing need to develop computational methods that allow the comparison of time series gene expression data with different length and sampling density, and have the capability to characterize more complicated temporal gene expression associations.

In this study, we developed TimeMeter, a statistical method and R package to assess temporal gene expression pattern similarity, and identify differentially progressing genes. TimeMeter uses the dynamic time warping (DTW) algorithm (15) to align two temporal sequences. Previous studies have shown that the DTW algorithm, which was originally designed for speech recognition, can be used to align gene pairs with nonlinearly related gene expression patterns which are sampled with different length and density (11,16–20). However, there are two shortcomings of using DTW to assess temporal similarity: (i) DTW gives optimal matches between two given time series sequences, regardless whether the temporal gene expression patterns are similar or not. Even for dissimilar patterns or patterns which are not comparable (e.g. one pattern only resembles a small fraction of another pattern), DTW still can return a best match by warping temporal sequences (10); (ii) when the number of time points is small, the likelihood of alignment arising by chance is high. To solve these problems, TimeMeter first post-processes a DTW alignment by truncating certain start or end points based on alignment patterns. This will result in a truncated alignment which represents the time frames from each time series that are comparable. Then it calculates four measurements that jointly assess gene pair temporal similarity: percentage of alignment for (i) query and for (ii) reference, respectively; (iii) aligned gene expression correlation; (iv) likelihood of alignment arising by chance. These four novel metrics in TimeMeter give temporal similarity assessments on different aspects, and the joint requirement of these four metrics will give a an assessment of the temporal pattern similarity.

For gene pairs with similar temporal patterns, TimeMeter partitions the temporal associations into separate segments via piecewise regression (21). The differential progression between gene pairs is calculated by aggregation of progression difference in each segment.

The axolotl, which is an important tetrapod model for research owing to its outstanding regenerative capabilities, is often compared with *Xenopus*, a tetrapod species with limited regenerative capabilities (22,23). These two species have already developed many unique genomic features during evolution (22,24). However, very little is known about their transcriptomic conservation in early embryonic development. In this study, we applied TimeMeter to two published datasets (25,26) to compare early embryonic development gene expression patterns (form stage 1 to stage 24) of these two species. We identified genes with similar temporal patterns (STP) between these two species in early embryonic development. Interestingly, we find a fraction of these STP genes undergo different progressions (one pattern is more advanced than the other in developmental progression from stage 1 to stage 24 which can be a result of dynamical speed differences, time shifts, or both), and they are enriched in functional groups, such as neural development, and smooth muscle cell proliferation. These results suggest that the measurement of differential progression (DP) may provide a novel feature that can characterize early developmental divergence between two species.

We next re-analyzed our previous study for comparing time series gene expressions of human embryonic stem (ES) cells and mouse epiblast stem (EpiS) cells during neural differentiation (13). We showed that TimeMeter significantly outperformed our previous method (13) for detecting STP genes. Further analysis suggests that these TimeMeter detected STP genes are naturally selected (under strong negative selection) during evolution, if compared to genes with dissimilar temporal patterns.

Finally, we used TimeMeter to detect STP genes between mouse digit regeneration and axolotl blastema differentiation. It is known that full appendage regeneration in the axolotl is due to the formation and differentiation of a heterogeneous pool of progenitor cells (blastema) at the site of amputation (27). The regeneration capability in human and mouse is mostly relegated to digit tips (28). Although studies suggest that blastema-like cells could be responsible for human/mouse limb regeneration (29), it is still largely unknown whether the limb regeneration process in human/mouse is similar to axolotl blastema differentiation. In this study, to examine the common mechanisms of limb regeneration, we amputated mouse digits and compared the post-amputation digit time series gene expression data with a published axolotl time series blastema differentiation RNA-seq dataset (30) via TimeMeter. Analysis of genes with similar temporal patterns in mouse digit regeneration and axolotl blastema differentiation reveals common gene groups for appendage regeneration which have potential implications in regenerative medicine.

## MATERIALS AND METHODS

### TimeMeter Algorithm: assessing temporal similarity

TimeMeter first uses the dynamic time warping (DTW) algorithm to align two time series gene expression vectors (a query and a reference; length may vary) via the R package (‘dtw’). However, one of the pre-assumptions of DTW algorithm is that the two sequences are comparable. For example, DTW assumes that (a) every aligned index from the first sequence must be matched with one or more indices from the other sequence, and vice versa; (b) the first and the last aligned indices from the first sequence must be matched with the first and the last indices from the other sequence, respectively (but it does not have to be its only match). These assumptions do not hold true for certain patterns, such as dissimilar patterns or patterns where one series only resembles a small fraction of another. For instance, if two temporal similar genes exhibit differential progression (e.g. time shift or different speed of dynamical change), when we use the same time window to compare gene expression patterns, certain time points (e.g. at start or at end) will be out of the matched time points boundary. DTW will make the start or the end points in one gene excessively duplicate to match the out of boundary time points in another gene. TimeMeter corrects the DTW aligned indices by truncating the first (*m* − 1) start time points in one gene if the first m time points can be aligned to the same start points in another gene, and terminating alignment (truncating the rest of time points) if DTW matches the last elements in any of the genes. This will exclude certain time points from alignment, and result in a truncated alignment. TimeMeter then calculates four measurements on the truncated alignment that jointly assess gene pair temporal similarity: percentage of alignment for (i) query and for (ii) reference, respectively; (iii) aligned gene expression correlation; (iv) likelihood of alignment arising by chance:

1. Percentage of alignment for query: the length of aligned time interval (after truncation) in query divided by total length of query time interval.
2. Percentage of alignment for reference: the length of aligned time interval (after truncation) in reference divided by total length of reference time interval.
3. Aligned gene expression correlation (Rho): it is calculated by the Spearman's rank correlation coefficient (Rho) for aligned gene expressions (after truncation). This measures how well the gene expression patterns correlate after alignment and truncation.
4. Likelihood of alignment arising by chance (*P*-value): To further rule out that the alignment is not due to a product of random chance, for each gene pair, TimeMeter shuffles the gene expression values of both query and reference separately 100 times. For each shuffling, the aligned gene expression Spearman correlation coefficient (Rho) (measurement 3) is calculated. TimeMeter assumes that the Rho from shuffling follows a Gaussian distribution with mean (*μ*) and standard deviation (*σ*). It calculates the *P*-value of likelihood of alignment arising by chance by lower-tail probability of Gaussian distribution (*μ*, *σ*^2^), assuming the aligned gene expression correlations between query and reference should be significantly higher than these shuffled temporal gene expressions.

These four metrics will give temporal similarity assessments on different aspects. In this study, we define similar temporal patterns (STP) as gene pairs where: (a) at least one temporal sequence (query or reference) has percentage of alignment >80%, and (b) no temporal sequence (query and reference) has percentage of alignment <50%. These two criteria assume that in the case that one pattern only resemble a fraction of another, the longer matched pattern should represent at least 80% of its original data, and the shorter matched pattern should represent at least half of its original data; and (c) Rho > 0.9 (the aligned gene expression values should be highly correlated) and (d) *P*-value < 0.05 (the likelihood of gene expression associations arising by chance should be <5%). These thresholds are used for identifying STP genes throughout this study.

### TimeMeter Algorithm: identifies differentially progressing genes

Given a STP gene pair (identified by previous step), TimeMeter scores the progression difference based on truncated alignment. For each query time point within a truncated alignment, TimeMeter groups and calculates the average corresponding aligned reference time. This will result in two variables: aligned query time as the independent variable and average aligned reference time as the dependent variable. Next, TimeMeter applies piecewise (segmented) regression to these two variables, and partitions them into separate segments. The breakpoints in piecewise regression are determined by the lowest Bayesian Information Criterion (BIC) via enumerating all *K* (*K* ≤ *N*) possible number of breakpoints (*N* = 10 in this study). For each segment, the slope of the regression measures the fold-change of the speed (query versus reference). A slope being greater or less than 1, indicates faster or slower dynamical changes, respectively. A slope equivalent or close to 1 is a special case in which the speed of dynamical change is the same or similar (time shift pattern). TimeMeter further merges adjacent segments if the absolute slope difference less than deltaSlope (we set deltaSlope = 0.1 for this study) by a linear regression, and recalculates the slope. This process is repeated until no adjacent segments have absolute slope difference less than deltaSlope. Then for each segment, TimeMeter calculates the area difference between under the segmented regression line and under the diagonal line, assuming that if the query and reference have no progression difference along time points, the aligned time points should follow the diagonal line (the aligned query time equals the aligned reference time). The extent of deviation from the diagonal line can be used to measure the progression difference. A progression advance score (PAS) is calculated by aggregation of area difference in each segment and normalized by total aligned time length (after truncation) in the query.

The PAS measures the absolute progression difference between two similar temporal pattern (STP) genes. For species with different paces of development (e.g. human versus mouse), the PAS may reflect the difference in development pace between the organisms. To investigate genes with ‘relative’ (‘unexpected’) progression difference (the differential progressions that cannot be explained by species-specific developmental paces), TimeMeter calculates the adjusted PAS. For each query time point, TimeMeter groups and calculates the median corresponding aligned reference time (after truncation) of all STP genes. This will result in two variables: the query time as the independent variable and the median aligned reference time of all STP genes as the dependent variable. Similar to calculating PAS for each gene pair, TimeMeter calculates a condition-specific progression advance score (c-PAS) that represent the overall progression difference between two conditions (e.g. species). For each STP gene, an adjusted PAS is calculated by PAS minus c-PAS, which represents the ‘unexpected’ progression difference (e.g., the differential progressions that cannot be explained by species-specific developmental paces) between two species.

### Data normalization and scaling

All gene expression values in this study were normalized and scaled from 0 to 1 using the following procedure: The normalization was performed by median-by-ratio normalization method (31). In case samples containing replicates, we merged replicates by calculating the average normalized gene expression values. Then we calculated log-transformed expression value as ‘log_10_ (normalized gene expression value + 1)’. We only included genes with significant changes in time series with at least 2-fold expression changes in time series, and scaled them from 0 to 1:

(1) $$\begin{equation*}{x_{i,\ scaled}} = \frac{{{x_i} - {x_{min}}}}{{{x_{max}} - {x_{min}}}}\end{equation*}$$

where ${x_{min}}$ and ${x_{max}}$ are minimal and maximal ‘log_10_ (normalized gene expression value + 1)’ of a gene in all conditions (e.g. time series).

### Adding noise in simulated data

To investigate how the data noise affects the *P*-values, we add different levels (*K*) of Gaussian noise *N*(0, 1) in the simulated data:

(2) $$\begin{equation*}{\rm{Query}} ( {{\rm{with\, noise}}}) = {\rm{Query}}( {{\rm{original}}}) + {{\rm{K}}^*}N ( {0,{\rm{ }}1})\end{equation*}$$

(3) $$\begin{eqnarray*}{\rm{Reference}}\,( {{\rm{with\, noise}}} ) = {\rm{Reference}}( {{\rm{original}}}) + {{\rm{K}}^*}N ( {0,{\rm{ }}1})\nonumber\\ \end{eqnarray*}$$

### Gene ontology analysis

Gene ontology (GO) enrichment analysis was performed using the R package (‘allez’) (32). For each GO enrichment analysis, the background (control) genes are matched with the target gene list using the same gene expression requirement (e.g. significant changes in time series) to avoid potential bias arises by gene expression difference. The *P*-values are further adjusted by Benjamini–Hochberg (BH) multiple test correction.

### Nonsynonymous substitution rate (d*N*) and the synonymous substitution rate (d*S*)

The d*N* and d*S* values between human and mouse protein coding genes were downloaded from Ensembl (v93) (33). We removed transcripts which have either d*N* or d*S* >1 to avoid potential paralogous transcript pairs (transcripts are duplicated after speciation, and the duplicated ones developed functions other than their ancestral one). These paralogous transcript pairs should be excluded for any natural selection analysis, because they are not comparable (34,35). For a gene with multiple transcripts, we selected the transcript with the smallest dS (36).

### Axolotl and *Xenopus* early developmental gene expression data

The axolotl early developmental gene expression data (transcripts per million (TPM)) were obtained from our previous study (26). The developmental stages were based on upon morphological staging (37). We excluded stage 40 from our analysis. This is because there is no data from stage 24 to stage 40 (a large sampling gap in time). Hence, we obtained TPMs from stage 1, 2, 3, 4, 5, 6, 7, 8, 9, 10, 11, 12, 14, 16, 19 and 24. We performed normalization and scaling on TPMs.

The *Xenopus* early development developmental gene expression data (Gaussian process lower median of ‘Transcripts per Embryo’, from stage 1 to stage 24) were obtained from the publication (25). This is a high sampling density dataset with 229 time points evenly distributed from stage 1 to stage 24. We divided the ‘Transcripts per Embryo’ by a factor of 1000 to match the magnitude change of TPMs (0–10^6^), and then performed normalization and scaling.

### Human embryonic stem (ES) cells and mouse epiblast stem (EpiS) cells differentiation gene expression data

The RNA-seq measured time series gene expression data (TPMs) on human embryonic stem (ES) cells and mouse epiblast stem (EpiS) cells during neural differentiation were obtained from our previous study (13). Human ES cells differentiation data contains 26 time points (from day 0 to day 42), and mouse EpiS cells differentiation contains 16 time points (from day 0 to day 21). Gene expression values (TPMs) were normalized and scaled from 0 to 1.

### Mouse digits regeneration data

1. *Surgical procedure*: All experiments were approved by the University of Wisconsin-Madison Institutional Animal Care and Use Committee. A total of 47 adult male C57Bl/6 mice (9–10 weeks old) were used for the study. Mice were subjected to hindlimb distal phalanx amputation to digit 3 (P3). For each amputation, mice were anesthetized, the hindlimb claw was extended, and the distal phalanx and footpad was sharply dissected. A regenerating distal phalanx was generated by amputating ≤33% of the P3. Skin wounds were allowed to heal without suturing. Mice were subjected to micro-computed tomography (microCT) one day prior to surgery and immediately after amputation to confirm ≤ 33% removal of the P3. Any digit that did not fall within the ≤ 33% amputation guideline was omitted from the study. Based on our criteria, 17 animals were removed from the study resulting in a final total of 30 mice. P3 mice were collected at 0, 3, 6, 12, 24 h, 3, 7, 14, 21 days. Each time point contained three mice, except day 7 (six mice). At the time of P3 collection, samples were immediately immersed in RNA later for 24 h at 4°C. The digits were then removed from RNAlater and stored at −80°C until performing RNA sequencing.
2. *MicroCT analysis*: Mice hindlimb paws were longitudinally imaged using microCT to assess digit regeneration. MicroCT provides the necessary resolution and contrast to measure digit length and volume used in the analysis. Imaging was performed using a Siemens Inveon microCT scanner, and analysis was conducted using Inveon Research Workplace General and 3D Visualization software (Siemens Medical Solutions USA, Inc., Knoxville, TN). All scans were acquired with the following parameters: 80 kV_p_, exposure time, 900 μA current, 220 rotation steps with 441 projections, ∼16.5-min scan time, bin by 2, 50 μm focal spot size, and medium magnification that yielded an overall reconstructed isotropic voxel size of 46.6 um^3^. Raw data were reconstructed with filtered back-projection and no down-sampling using integrated high-speed COBRA reconstruction software (Exxim Computing Corporation, Pleasanton, CA, USA). Hounsfield units (HU), a scalar linear attenuation coefficient, was applied to each reconstruction to permit inter-subject comparisons. Three-dimensional images were segmented using a minimum pixel intensity of 300 HU, and a maximum intensity of 3168 HU to represent bone density. After the region of interest was defined, the P3 volume was calculated. Sagittal length of the digits was also obtained by measuring twice from the distal tip to proximal edge of the P3 bone. Two researchers who were blinded to one another's measurements independently conducted analyses, and their results were averaged.
3. *RNA-seq*: Total RNAs were isolated from tissues using trizol (ThermoFisher #15596018) and chloroform phase separations followed by the RNeasy mini protocol (Qiagen #74106) with optional on-column DNase digestion (Qiagen #79254). One hundred nanograms of total RNA was used to prepare sequencing libraries using the LM-Seq (Ligation Mediated Sequencing) protocol (38). RNAs was selected using the NEB Next Poly A+ Isolation Kit (NEB #E7490S/L). Poly A+ fractions were eluted, primed, and fragmented for 7 min at 85°C. First stand cDNA synthesis was performed using SmartScribe Reverse Transcriptase (Takara Bio USA #639538) and RNA is then removed. cDNA fragments were purified with AMpure XP beads (Beckman Coulter #A63881). The 5′ adapter was ligated and 18 cycles of amplification were performed. These final indexed cDNA libraries were quantified, normalized, multiplexed, and run as single-end reads for 65 bp on the HiSeq 2500 (Illumina, San Diego, CA, USA).
4. *Mapping RNA-seq reads and calculating gene expressions*: Reads were mapped to the mouse genes (Ensembl v75) using Bowtie (v0.12.8) (39) allowing up to two mismatches and a maximum of 200 multiple hits. The gene expected read counts and TPMs were estimated by RSEM (v1.2.3) (40). TPMs were median-by-ratio normalized (31), and replicates were merged via calculating average normalized TPMs.
5. *Data access*: The RNA-seq raw data (fastq files) and the processed data (TPMs and expected counts) for the mouse digit regeneration data have been submitted to GEO with accession number GSE130438.

### Axolotl blastema cell differentiation data

The raw axolotl blastema cell differentiation RNA-seq reads were obtained from the previous study (30). To compare with mouse digit regeneration data, we re-processed axolotl raw reads via mapping axolotl contigs to mouse gene annotations. We obtained axolotl transcriptome assembled contigs from a prior study (41). We used cd-hit-est (v4.6) (42) with parameter ‘-c 1’ to remove shorter contigs with 100% identity with aligned longer contigs. These non-redundant contigs were mapped to Ensembl mouse proteins (v85) by BLASTX (v2.2.18). Contigs were assigned to mouse proteins by taking the best BLASTX hit with *E*-value < 10^−5^.

We used Bowtie (v0.12.1) (39) to map the axolotl blastema cell differentiation RNA-seq reads against all non-redundant contigs. Quantification of each contig was performed by RSEM (v1.2.3) (40). For each sample, the expected fragment counts for each contig (as computed by RSEM), were then converted to comparative transcript counts by summing the fragment counts of contigs mapped to the same transcript. Similarly, gene-level counts were obtained by summing the fragment counts of transcripts that were annotated with the same gene symbol. Relative abundances, in terms of TPMs, for genes were computed by first normalizing each gene's fragment count by the sum of the ‘effective lengths’ (weighted average of contigs length based on contigs abundance) of the contigs mapped to that gene and then scaling the resulting values such that they summed to one million over all genes. TPMs were normalized and scaled from 0 to 1.

## RESULTS

### Overview of TimeMeter method and simulation studies

**Figure 1.**
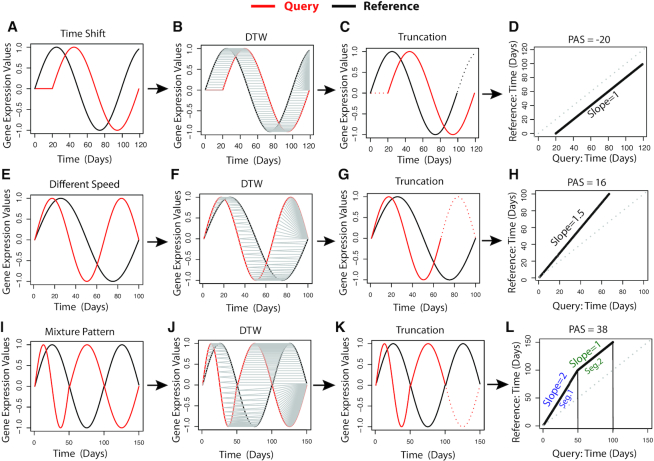
Illustration of TimeMeter by simulated high density discrete data. (**A**–**D**) Time shift pattern. (**E–H**) Different speed of dynamical change pattern. (**I–L**) Mixed pattern: the query has a 2-fold faster in dynamical change compared to the reference for the first 50 days, but after the first 50 days, the query has the same speed of dynamical change with the reference. TimeMeter uses DTW to align gene expression values, and then prunes excessively duplicated start or end points aligned indices, and truncates corresponding time points (C, G and K; dashed lines). TimeMeter applies piecewise (segmented) regression to aligned time points (after truncation), and partitions them into separate segments if more than one pattern is detected, such as figure (I). A progression advance score (PAS) is calculated by aggregation of area difference in each segment and normalized by total aligned time length (after truncation) in query.

Figure 1 shows the simulated data. Given a pair of time series gene expression data (Figure 1A, E and I), TimeMeter uses the DTW algorithm to align them (Figure 1B, F and J; gray lines indicate aligned indices). TimeMeter then post-processes the DTW alignment by truncating certain start or end points based on alignment patterns (Figure 1C, G and K; dashed lines indicate time points removed by truncation; see Materials and Methods). After truncating the temporal pattern (Figure 1C, G and K; solid lines), TimeMeter calculates four metrics that jointly assess gene pair temporal similarity: percentage of alignment for (i) query and for (ii) reference, respectively; (iii) aligned gene expression correlation; (iv) likelihood of alignment arising by chance (see Materials and Methods).

In the example shown in Supplementary Figure S1A, the temporal pattern of the query is delayed by 5 days (a time shift pattern) with respect to the reference. If we use different time windows, aligning days 5 through 100 of the query to days 0 through 95 of the reference, the query and the reference will be perfectly matched. However, since our data comprises the same observation window (from day 0 to day 100) for both the query and the reference, certain time points (e.g. the first 5 days in the query and the last 5 days in the reference) fall outside the shifted time interval overlap. TimeMeter corrects this by analyzing the aligned indices at the start and the end of the alignment, and removing the first 5 days from the query (Supplementary Figure S1C; red dashed lines) and the last 5 days from the reference (Supplementary Figure S1C; black dashed lines). Hence, the percentage of alignment for both query and reference is 95%. After truncation, TimeMeter calculates the Spearman's rank correlation coefficient (Rho) of the remaining aligned (matched) gene expression values. In this case, the Rho is 1, indicating the query and reference patterns are perfectly matched (after truncation). TimeMeter then estimates the likelihood of alignment arising by chance (*P*-value = 3.5e–18) based on shuffling of the original temporal gene expression data (see Materials and Methods). Given the same time window, if the query and the reference are shifted by a longer time (e.g. 15 days or 30 days) (Supplementary Figure S1e and i), more time points will be truncated, resulting in a lower percentage of alignment for both the query and the reference (Supplementary Figure S1G and K).

When we use the same time window to compare two temporal patterns with different dynamical speed (one is faster in dynamical changes than the other, rather than simply offset by a specific time interval) (Supplementary Figure S2), TimeMeter will truncate the dynamically faster pattern, indicating that the dynamically slower pattern only resembles a fraction of the dynamically faster pattern. Given the same time window, an increase of the difference of dynamical speed between the query and the reference will result in more truncation for the dynamical faster pattern (Supplementary Figure S2C, G and K).

**Figure 2.**
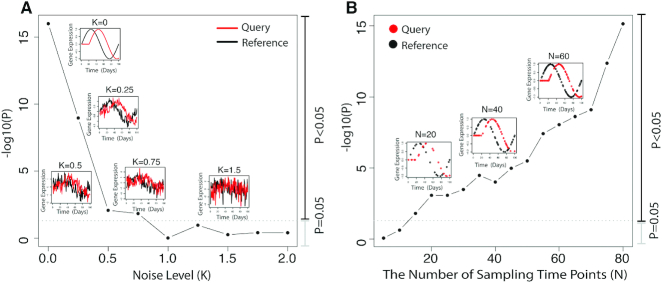
Simulation study of how the data noise and sampling density will affect *P*-values in TimeMeter. The query and the reference have a time shift pattern (simulated discrete time series data). (**A**) Increasing the noise level will decrease the power to detect the pattern associations. (**B**) A higher sampling density will increase the power to detect the pattern associations.

We further investigated how the data noise and the sampling density affect the *P*-values. As shown in Figure 2, given a truly correlated time shift pattern, a rising in noise level will decrease the power to detect the pattern associations (Figure 2A) while a higher sampling density will increase the power to detect the pattern associations (Figure 2B). The same trend can also be seen in simulations on different dynamical speed patterns (Supplementary Figure S3).

For gene pairs with similar temporal patterns (STP) (Materials and Methods), TimeMeter applies piecewise (segmented) regression to aligned time points (after truncation), and partition them into separate segments. For example, as shown in Figure 1i, the query and reference exhibit a mixed pattern of matching where the query has a 2-fold faster dynamical change compared to the reference for the first 50 days, but after the first 50 days, the query has the same speed of dynamical change with the reference. TimeMeter partitions the aligned time points (after truncation) (Figure 1K) into two segments (Figure 1l) with slope = 2 and slope = 1, respectively. In segment 1 (slope = 2), the first 50 days in query are aligned to the first 100 days in reference, indicating a 2-fold faster dynamical change in the query. In segment 2 (slope = 1), the days (51–100) in query are aligned to the days (101–150) in reference, indicating that the query and reference have the same speed of dynamical change (slope = 1). Segment 2 represents a time shift in this case owing to the prior difference of speed in dynamical change in segment 1. For each segment, we assume that if the query and the reference have no progression difference, the aligned time points should follow the diagonal line (the aligned query time equals the aligned reference time). Hence, for each segment, TimeMeter calculates the area difference between the segmented regression line and the diagonal line, assuming that the area difference measures the progression difference. A progression advance score (PAS) is calculated by aggregation of area difference in each segment and normalized by total aligned time length (after truncation) in query (see Materials and Methods). The time shift pattern is a special case where the PAS equals the shifted time in query (Figure 1D and Supplementary Figure S1). For other patterns, such as different speed of dynamical change (Figure 1E and Supplementary Figure S2) and mixed pattern (e.g. a mixture of time shift and different speed of dynamical change patterns) (Figure 1I), PAS measures the overall progression difference between the query and the reference overtime.

### Comparison of axolotl and *Xenopus* during early embryonic development

**Figure 3.**
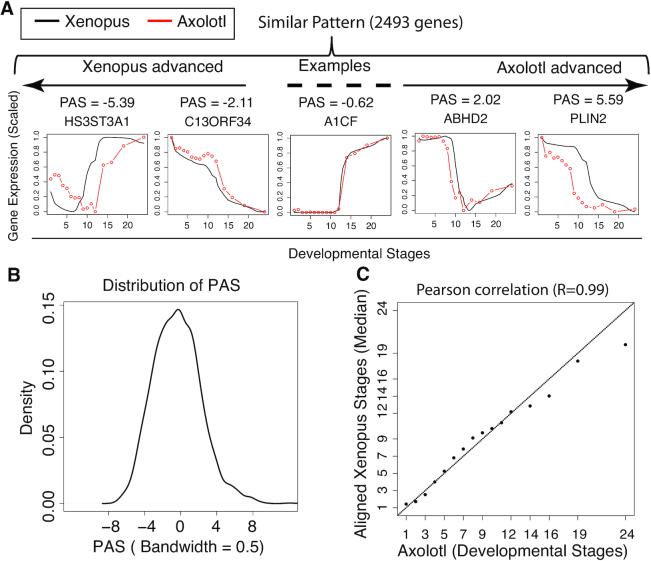
Comparison of axolotl and *Xenopus* during early embryonic development. TimeMeter detects 2493 genes with similar temporal patterns (STP) between these two species. (**A**) Examples of STP genes with different PAS. (**B**) PAS distribution of STP genes. (**C**) Correlation between axolotl developmental stages and aligned *Xenopus* stages of STP genes.

To compare temporal gene expression patterns in early embryonic development between axolotl and *Xenopus*, we obtained time series gene expression values (from stage 1 to stage 24 based upon morphological staging) from two publications (25,26). After data normalization, filtering and scaling (Materials and Methods), we obtained 10 252 genes for downstream analysis. We applied TimeMeter to these genes, and detected 2493 (24.31%) STP genes between these two species (Supplementary Table S1). These STP genes are enriched in a broad spectrum of gene ontology (GO) terms (BH adjusted *P*-value (*P*.adj) < 0.05) (Supplementary Table S2) (e.g. nuclear-transcribed mRNA catabolic process, nonsense-mediated decay, rRNA processing, translational initiation and others). Interestingly, among enriched GO terms, there are 17 terms related to development (e.g. blood vessel development) and 10 terms related to morphogenesis terms (e.g. embryonic organ morphogenesis) (Table 1). We further calculated the PAS for each STP gene to investigate developmental progression differences (Figure 3A). As shown in Figure 3B, the PAS distribution is symmetric centering close to 0. Furthermore, the axolotl developmental stages are highly correlated with the temporal aligned *Xenopus* stages (*R* = 0.99, Pearson correlation coefficient; Figure 3C). This suggest that although the divergence time between axolotl and *Xenopus* was around 290 million years ago (MYA) (43), the dynamical change patterns of the transcriptome in early developmental stages is highly conserved.

**Table 1.** Enriched development and morphogenesis related GO terms (*P*.adj < 0.05) for genes with similar temporal patterns during early embryonic development between axolotl and *Xenopus*.

ID	Term (BP)	*P*.adj
**Enriched Development Related Terms**		
GO:0001568	Blood vessel development	4.60e–03
GO:0048568	Embryonic organ development	5.37e–03
GO:0060065	Uterus development	1.06e–02
GO:0009790	Embryo development	1.29e–02
GO:0043009	Chordate embryonic development	1.51e–02
GO:0001944	Vasculature development	1.72e–02
GO:0048565	Digestive tract development	1.72e–02
GO:0048566	Embryonic digestive tract development	1.72e–02
GO:0001823	Mesonephros development	1.98e–02
GO:0009792	Embryo development ending in birth or egg hatching	1.98e–02
GO:0072358	Cardiovascular system development	2.30e–02
GO:0021954	Central nervous system neuron development	2.55e–02
GO:0001656	Metanephros development	2.79e–02
GO:0001945	Lymph vessel development	3.13e–02
GO:0048706	Embryonic skeletal system development	3.13e–02
GO:0055123	Digestive system development	3.13e–02
GO:0021884	Forebrain neuron development	4.21e–02
**Enriched Morphogenesis Related Terms**		
GO:0035239	Tube morphogenesis	1.63e–03
GO:0048562	Embryonic organ morphogenesis	2.17e–03
GO:0048598	Embryonic morphogenesis	2.58e–03
GO:0009887	Animal organ morphogenesis	4.56e–03
GO:0048514	Blood vessel morphogenesis	1.46e–02
GO:0061138	Morphogenesis of a branching epithelium	2.55e–02
GO:0048754	Branching morphogenesis of an epithelial tube	3.54e–02
GO:0001763	Morphogenesis of a branching structure	3.83e–02
GO:0048646	Anatomical structure formation involved in morphogenesis	3.90e–02
GO:0048557	Embryonic digestive tract morphogenesis	4.21e–02

**Figure 4.**
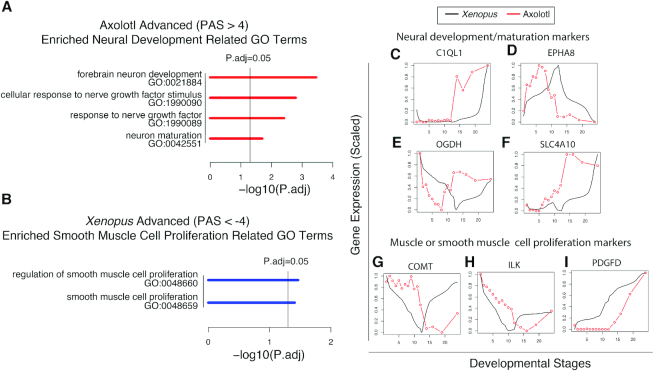
Differential progression genes (|PAS| > 4) between axolotl and *Xenopus* during early embryo development. (**A**) Enriched neural development related GO terms in Axolotl advanced genes. (**B**) Enriched muscle or smooth muscle related GO terms in *Xenopus* advanced genes. (**C–F**) Examples of Axolotl advanced neural development/maturation markers. (**G–I**) Examples of *Xenopus* advanced muscle or smooth muscle markers.

PAS measures the overall progression difference between the axolotl and the *Xenopus* during development. Visually, one can observe a noticeable progression difference if |PAS > 2| (Figure 3A). Actually, if the progression difference is only due to a time shift, the PAS equals the shifted time in query (Figure 1D and Supplementary Figure S1, simulated data). If we use a more stringent PAS cutoff (|PAS| > 4), there are 126 axolotl advanced genes (PAS>4) and 192 *Xenopus* advanced genes (PAS←4), respectively. Interestingly, for axolotl advanced genes (PAS > 4), several well-known key neural development genes are in list, such as C1QL1 (44), EPHA8 (45), OGDH (46) and SLC4A10 (47) (Figure 4C–F). For *Xenopus* advanced genes (PAS < −4), several well-known muscle or smooth muscle cell proliferation genes are in the list, such as COMT (48), ILK (49) and PDGFD (50) (Figure 4G–I). We performed GO enrichment analysis for axolotl and *Xenopus* advanced genes, respectively (Supplementary Table S3). Among the 11 enriched GO terms (*P*.adj < 0.05) in axolotl advanced genes (e.g. bicarbonate transport, forebrain neuron development, one-carbon metabolic process, and others), four neural development related terms are enriched (Figure 4A). Among the 14 enriched GO terms (*P*.adj < 0.05) in *Xenopus* advanced genes (e.g. regulation of protein sumoylation, regulation of endoplasmic reticulum unfolded protein response, and others), two muscle cell proliferation related terms are enriched (Figure 4b). If we use an even more stringent cutoff (|PAS| > 5) to define differentially progressing genes, three out of four neural development related terms in genes with PAS > 4 cutoff are also enriched in genes with PAS > 5 cutoff (Supplementary Figure S4A). The two enriched muscle cell proliferation related terms in genes with PAS < −4 cutoff are also enriched in genes with PAS < −5 cutoff (Supplementary Figure S4B). These results suggest that a fraction of functional distinct temporal similar genes undergo different progressions in the axolotl compared to *Xenopus* during early embryo development.

### Reanalysis of human and mouse time series gene expression comparison during neural differentiation

**Figure 5.**
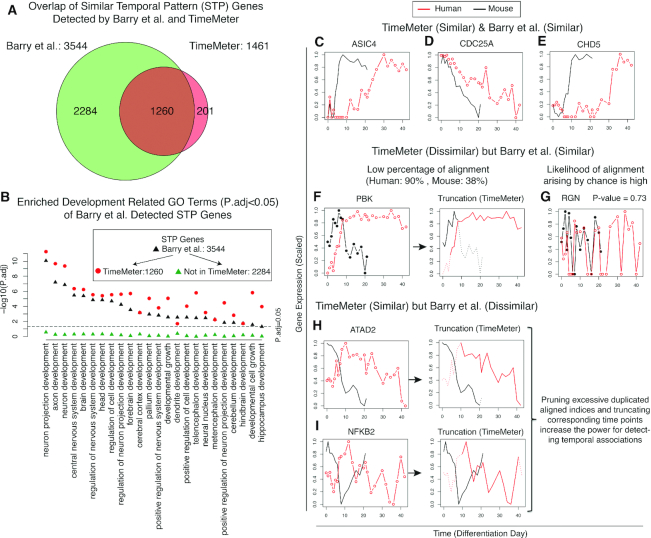
Comparison of TimeMeter and Barry *et al.* for detecting genes with similar temporal patterns (STP) between human ES (from day 0 to day 42) and mouse EpiS cells (from day 0 to day 21) during neural differentiation. (**A**) Overlap of STP genes detected by Barry *et al.* and TimeMeter. (**B**) TimeMeter significantly increases the specificity for detecting STP genes. Barry *et al.* detected STP genes are enriched in 24 development related GO terms (*P*.adj < 0.05) (black triangle). None of these development related GO terms is enriched (*P*.adj < 0.05) in Barry *et al.* only gene list. In contrast, 20 out of 24 development related GO terms showed noticeable increased statistical significance for 1260 STP genes which were also detected by TimeMeter. (**C**–**E**) Examples of STP genes detected by both TimeMeter and Barry *et al.* (**F**, **G**) Examples of STP genes which were detected only by Barry *et al.* but not by TimeMeter. (**H**, **I**) STP genes which were detected only by TimeMeter but not by Barry *et al.*

Our previous study compared the transcriptomic dynamical changes between human embryonic stem (ES) cells (from day 0 to day 42) and mouse epiblast stem (EpiS) cells (from day 0 to day 21) during neural differentiation (13). In Barry *et al.*, a DTW algorithm coupled with a set of statistical methods (13) was used to detect 3544 STP genes between the human and mouse time series. We used TimeMeter to reanalyze this dataset (Materials and Methods). As shown in Figure 5A, TimeMeter detects 1461 STP genes between human and mouse (Supplementary Table S4). The majority of TimeMeter detected STP genes (1260, 86.24%) can also be detected by our previous study (13). Figure 5C–E are examples of STP genes which are detected by both TimeMeter and Barry *et al.* However, a large portion of Barry *et al.* detected STP genes (2284, 64.45%) cannot be detected by TimeMeter.

To investigate whether TimeMeter substantially increased the specificity for detecting STP genes, we decomposed the Barry *et al.* detected 3544 STP genes into two lists: (i) 1260 STP genes, which are also detected by TimeMeter (referred as ‘Both’) and (2) 2,284 STP genes, which are only detected by Barry *et al.* (not by TimeMeter; referred as ‘Barry *et al.* only’), and performed the following analysis:

Firstly, we performed GO enrichment analysis on ‘Both’ and ‘Barry *et al.* only’ STP genes, respectively. There are more enriched GO terms in ‘Both’ than in ‘Barry *et al.* only’ STP genes. As shown in Supplementary Figure S5, 1,260 ‘Both’ STP genes are enriched in 474 GO terms (*P*.adj < 0.05), while 2284 ‘Barry *et al.* only’ STP genes are only enriched in 108 GO terms. The ‘Both’ STP genes enriched GO terms covers the majority of ‘Barry *et al.* only’ enriched GO terms but not vice versa. As shown in Supplementary Figure S5, a large portion (60.2%, 65/108) of ‘Barry *et al.* only’ enriched GO terms are also enriched in ‘Both’ STP gene sets. However, only 13.7% (65/474) of ‘Both’ STP gene sets enriched GO terms are also enriched in ‘Barry *et al.* only’ STP gene sets. For enriched GO terms, the majority of ‘Both’ STP genes (73.4%, 925/1260) are driving genes (genes that drive the enrichment GO terms) while only around half (49.9%, 1139/2248) of ‘Barry *et al.* only’ STP genes are GO driving genes. The GO enrichment analysis suggests that TimeMeter did not randomly pick up a subset of STP genes from the Barry *et al.* STP gene sets (Figure 5a). Instead, TimeMeter detected STP genes that tend to be associated with specific biological functions. In contrast, the ‘Barry *et al.* only’ STP gene sets are more randomly distributed to all GO terms (e.g., less enriched GO terms, smaller percentage of GO driving genes).

Secondly, the neuron or morphogenesis development related GO terms are top enriched specifically in ‘Both’ gene sets (enriched in ‘Both’ but not in ‘Barry *et al.* only’). The STP genes are based on comparing mouse epiblast stem (EpiS) cells differentiated to neural cells with human embryonic stem (ES) cells differentiated to neural cells, and thus it would be expected to find neuron morphogenesis and neuron development terms to be enriched. The ‘Barry *et al.* only’ top 5 enriched GO terms are not directly related to neural development.

Thirdly, we investigated whether our previous reported development related functions of STP genes were in fact driven by a subset of ‘real STP genes’. We recalculated the GO enrichments in the Barry *et al.* STP gene set. Among the enriched (*P*.adj < 0.05) GO terms for the 3544 STP genes from Barry *et al.*, there are 24 terms related to development (Figure 5B). As shown in Figure 5B, none of the original enriched development related GO terms are enriched (*P*.adj < 0.05) in the Barry *et al.* only gene list. In contrast, 20 out of 24 development related GO terms showed noticeably higher statistical significance for 1260 STP genes identified by both methods (Figure 5B). This suggests that TimeMeter significantly increased the specificity for detecting STP genes.

Fourthly, we integrated the knowledge of human-mouse Carnegie stage equivalents (*in utero*) to evaluate TimeMeter and Barry *et al.* methods. It is technically challenging to directly use traditional correlation analysis to evaluate TimeMeter and Barry *et al.* methods, because these two datasets have different numbers of time points. However, it is a widely accepted notion that the Carnegie stage progression (developmental chronology) during gestation can be directly comparable between human and mouse (51,52). For example, the human embryonic day 14 is equivalent to mouse embryonic day 6 *in utero* based on the external and/or internal morphological development of the embryo (UNSW Embryology website: https://embryology.med.unsw.edu.au/embryology/index.php/Carnegie_Stage_Comparison). Hence, if we use a subset of time points to reconstruct a human-mouse time points pair *in vitro* (Barry *et al.* datasets) (e.g. human day 16 matches to mouse day 7, human day 22 matches to mouse day 10) that resemble human-mouse Carnegie stage equivalents (*in utero*), the newly constructed gene expression pair will not only have the same number of time points but also will have been adjusted by the differentially developmental paces, since the time points in human and mouse are matched to Carnegie stage equivalents. Therefore, we can apply traditional correlation analysis to evaluate TimeMeter and Barry *et al.* methods. We transposed Barry *et al.* (*in vitro*) days to embryonic day equivalents (*in utero*) (a detailed explanation for this transposition can be found in Barry *et al.* (13)), and selected a subset of time points, and then reconstructed the human-mouse time points pair that can match human-mouse Carnegie stage equivalents (*in utero*) (Supplementary Figure S6a). As shown in Supplementary Figure S6B, the ‘Both’ STP gene pairs have overall better correlations than the ‘Barry *et al.* only’ STP genes (*P* = 9.67e–47, 1-sided Kolmogorov–Smirnov test). This analysis suggests that TimeMeter significantly increases the specificity for detecting STP genes.

Figure 5F and G shows expression patterns for two STP genes that were detected only by Barry *et al.* but not by TimeMeter. Figure 5F is an example where one temporal pattern only matches a small fraction of another pattern. Hence, after truncation, the percentage of alignment is very low (Figure 5F, dashed lines indicate removed time point by truncation). Figure 5G is an example with a high likelihood of a comparable temporal pattern association arising by chance.

Only 201 STP genes were detected by TimeMeter but not by Barry *et al.* (Figure 5A). On inspection, we found that TimeMeter's truncation feature appears to increase the sensitivity for detecting temporal associations (e.g. Figure 5H and I) while the thresholding metrics, such as the percentage of alignment, maintain specificity.

**Figure 6.**
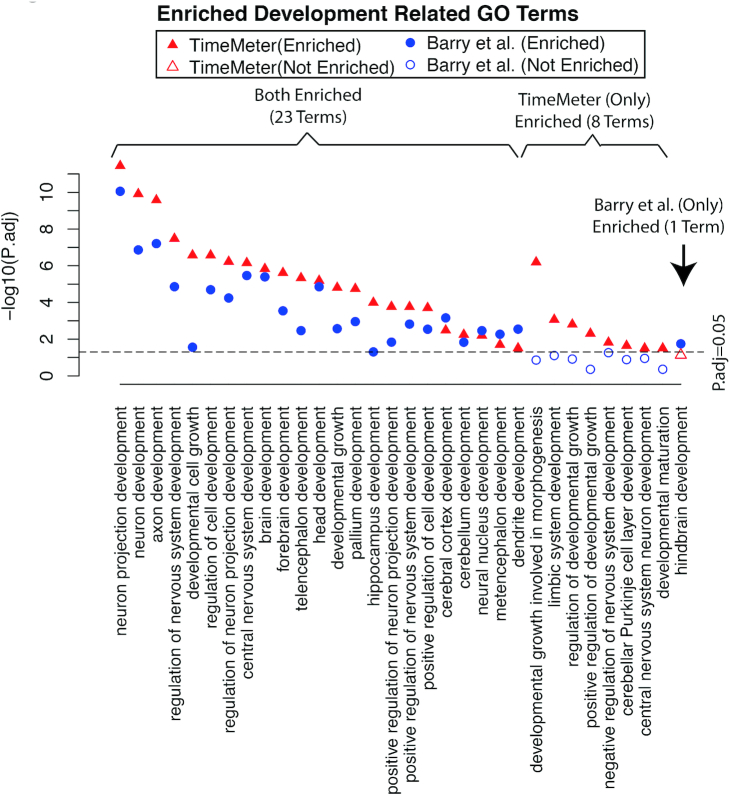
The increased specificity for detecting genes with similar temporal pattern (STP) of TimeMeter is not at the cost of losing sensitivity. There are 32 development related GO terms are enriched (*P*.adj < 0.05) in either TimeMeter or Barry *et al.* detected STP genes between human ES and mouse EpiS during neural differentiation. There are eight terms specifically enriched in TimeMeter detected STP genes (but not enriched in Barry *et al.* list) while there is only one term marginally enriched in Barry *et al.* list (but not enriched in TimeMeter list).

To further investigate whether the increased specificity of TimeMeter is at the cost of decreased sensitivity, we performed separate GO enrichment analysis on the STP gene sets from TimeMeter and from Barry *et al.*, respectively. As shown in Figure 6, 32 development related GO terms are enriched (*P*.adj < 0.05) in either the TimeMeter list or in the Barry *et al.* list. Of the 23 terms found in both lists, 19 are more significantly enriched in the TimeMeter list than in the Barry *et al.* list. Another eight terms are specifically enriched in TimeMeter STP gene list, while only one term (hindbrain development) is close to the level of significance (*P*.adj = 0.07) only in Barry *et al.* list. These results suggest that TimeMeter's increased specificity is not at the cost of decreased sensitivity.

**Figure 7.**
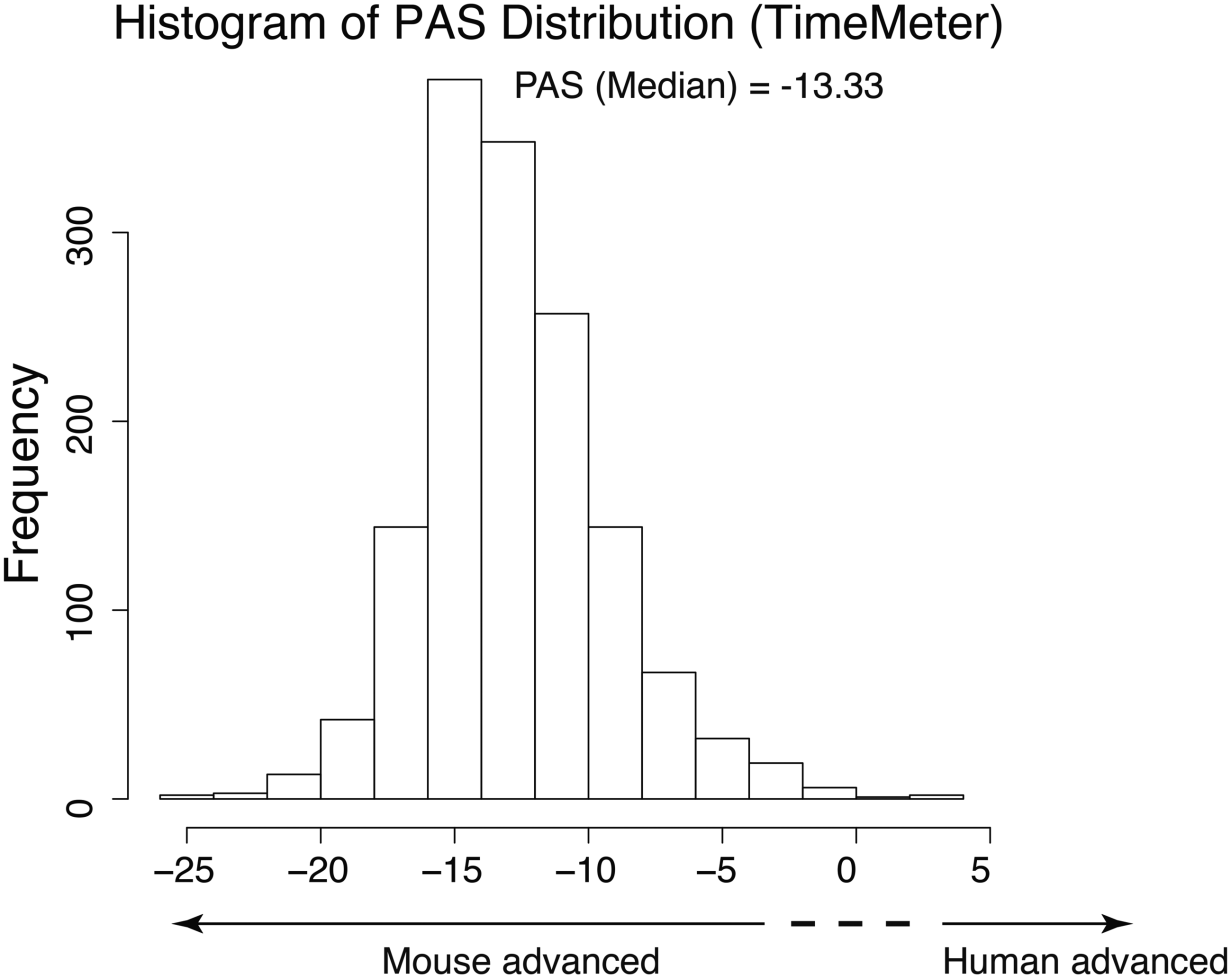
PAS distribution of genes with similar temporal patterns (STP) between human ES and mouse EpiS during neural differentiation.

We next calculated the PAS distribution for TimeMeter detected STP genes. As shown in Figure 7, the median PAS is −13.33, and 99.79% of PAS are <0. These results indicate that most of the mouse genes have more advanced progressions than human during neural cell differentiation, which is consistent with our previous study (13). The PAS measures the ‘absolute’ progression difference between two STP genes. It is an unbiased way to compare the progression difference for species of unknown developmental paces. However, the human and the mouse have different speed of developmental paces. To measure the ‘relative’ (‘unexpected’) progression difference (the differential progressions that cannot be explained by species-specific developmental paces), we also calculated an adjusted PAS value (Materials and Methods) for each STP gene (Supplementary Table S4). As shown in Supplementary Figure S7, the adjusted PAS distribution is symmetric centering close to 0. The unadjusted PAS (representing the absolute progression differences) and the adjusted PAS (representing the relative progression differences) in TimeMeter provide two separate measurements to characterize the progression difference for a STP pair.

**Figure 8.**
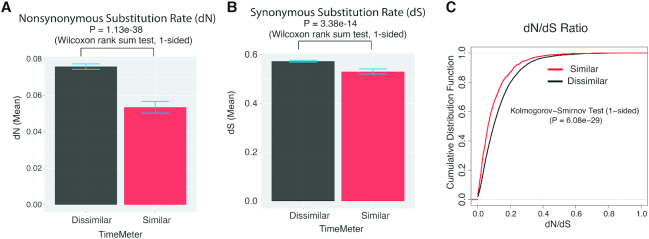
Nonsynonymous and synonymous substitution rates for temporally similar and dissimilar genes between human ES and mouse EpiS during neural differentiation. (**A**) Nonsynonymous substitution rate (d*N*). (**B**) Synonymous substitution rate (d*S*). (**C**) dN/dS ratio.

During evolution, the ratio of the nonsynonymous substitution rate (d*N*) and the synonymous substitution rate (d*S*) estimates the balance between neutral mutations, purifying selection and beneficial mutations. A d*N*/d*S* ratio is significantly <1 indicates that the gene is under strong purifying selection (acting against change). We asked whether the genes, which have conserved (similar) temporal patterns between human and mouse during neural development, are naturally selected. We examined this by comparing the d*N*/d*S* ratio between genes identified by TimeMeter with similar and dissimilar temporal patterns. As shown in Figure 8, the d*N*/d*S* ratio is significantly lower in STP genes than in dissimilar genes (*P* = 6.08e–29, 1-sided Kolmogorov–Smirnov test), indicating that the STP genes are naturally selected (against protein functional changes) during evolution.

### Identification of genes with similar temporal patterns during mouse digit regeneration and axolotl blastema differentiation

**Figure 9.**
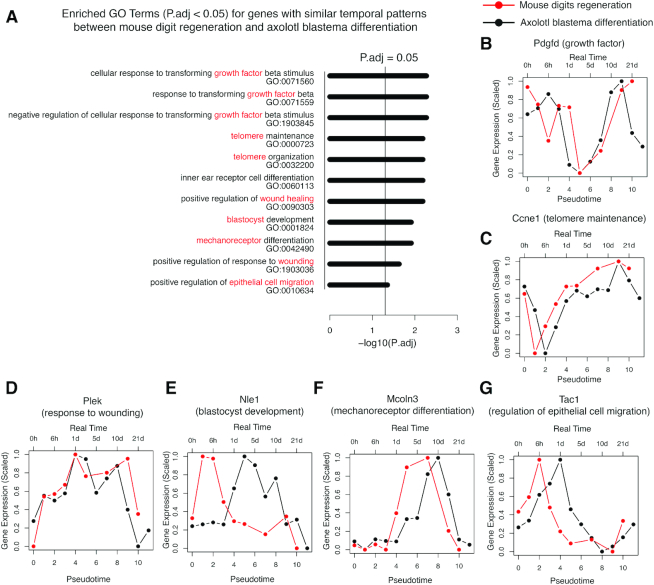
Genes with similar time-order patterns (STP) during mouse limb regeneration and axolotl blastema differentiation. (**A**) Enriched GO terms. (**B–G**) Examples of STP genes.

Full appendage regeneration in the axolotl is due to the formation and differentiation of blastema cells at the site of amputation (27). However, regeneration capability in human and mouse is mostly relegated to digit tips (28). To investigate any similarity between the human/mouse digits regeneration and the axolotl blastema differentiation, we produced a mouse digit regeneration RNA-seq time series dataset (Methods), and compared it with an axolotl blastema differentiation RNA-seq time series dataset (30). We reprocessed the axolotl RNA-seq data via mapping assembly contigs to mouse annotations to compare with our mouse post-amputation digit gene expression data (Methods). Due to experimental limitations (e.g. cost), both our mouse limb regeneration data and the published axolotl blastema differentiation data contained large sampling gaps between certain time points (uneven sampling). TimeMeter can be applied to scenarios with different sampling densities, but not uneven sampling in time (Supplementary Figure S5; see Discussions). To solve this problem, we replaced the real time (hours, days) with the pseudo-time (time order), aiming to detect temporally similar genes in time order. TimeMeter detects 38 STP genes during mouse limb regeneration and axolotl blastema differentiation (Supplementary Table S5). GO analysis indicates that these STP genes are enriched in 11 terms (*P*.adj < 0.05; Figure 9A), and most of them have relationship to ‘growth factor’, ‘telomere’, ‘wound healing’ etc. It is known that platelet-derived growth factor (PDGF) plays a pivotal role in bone and vascular smooth muscle formation via increasing the healing cell populations, facilitating to develop new blood vessels, and debriding the wound site for continued repair and bone regeneration (53). As shown in Figure 9B, Pdgfd (platelet-derived growth factor D) gene, which is a member of PDGF family, has similar patterns in mouse limb regeneration and axolotl blastema differentiation. Among the enriched GO terms, telomere maintenance and organization terms are particular interesting (Figure 9A). Several studies have shown that telomeres are critical for cardiovascular (54) and kidney (55) tissue repair and regeneration. Our study further highlights the potential general role of telomeres for regeneration. Examples of STP genes related to telomere maintenance, response to wounding, blastocyst development, mechanoreceptor differentiation and regulation of epithelial cell migration are shown in Figure 9C–G, respectively.

## DISCUSSION

Comparative time series gene expression analysis is a powerful tool that allows us to characterize time dependent changes of development, evolution, aging, disease progression, and cancer prognosis. While existing studies largely focused on identifying time dependent differentially expressed genes (1–7,56–58) and characterizing time shift patterns (9–11), additional computational tools are needed to enhance our capability to capture more complicated temporal gene expression associations. The DTW algorithm, which was originally designed for speech recognition (e.g. coping with different speaking speeds), has been used to align non-linearly associated temporal gene expression patterns (12,13,17,19,20). However, DTW will give an optimal match between two given time series sequences, regardless of whether the temporal gene expression patterns are similar or not (10). Although the distance or correlations calculated by DTW aligned sequences can partially measure the pattern similarity (17), the alignment could contain errors for dissimilar patterns or patterns where one resembles only a small fraction of the other. Moreover, for genes pairs with limited temporal sampling density or containing a high amount of noise, the likelihood of DTW alignment arising by chance is very high. To solve these issues, TimeMeter post-processed DTW aligned indices, and calculated four metrics that jointly assess gene pair temporal similarity. TimeMeter significantly outperformed our previous method (13) which was based on standard DTW to detect STP genes.

We have developed an R package (‘TimeMeter’). TimeMeter can be applied to compare time series gene expression data allowing query and reference samples to be in different time windows (Supplementary Figure S8A–E) and different sampling densities (Supplementary Figure S8B). TimeMeter does not allow uneven sampling (large gaps in time). Large sampling gaps within experimental data may potentially cause bias in several steps, such as DTW alignment (e.g. duplicated alignment due to missing data) and segmented regression (e.g. confounding break point determination). An alternative approach is to transform real time (e.g. 0, 3, 6, 12, 24 h, 3, 7, 14, 21 days) to pseudo-time (time order) (e.g. 0, 1, 2, 3, 4, 5, 6, 7, 8). The pseudo-time is evenly distributed (each is separated by 1 unit) (Supplementary Figure S8F). In this scenario, TimeMeter will aim to detect ‘time-order similar genes’ (Figure 9B–G).

Axolotl and *Xenopus* diverged about 290 million years ago (MYA) (43), and very little is known about their transcriptomic conservation in early embryo development. We found 2493 (24.31%) variant genes showed similar temporal patterns between these two species. These STP genes are enriched in a broad spectrum of functional groups important for development and morphogenesis (Table 1 and Supplementary Table S1). These findings suggest that a substantial number of developmental related genes are transcriptionally conserved in early embryonic development between these two species across 290 million years of evolution. We further characterized these STP genes by calculating the progression advance scores (PAS). Interestingly, the genes showed different progressions between Axolotl and *Xenopus* (e.g. |PAS| > 4) are enriched in function groups which are important for neural and muscle development, indicating that the measurement of differential progression provides a novel feature in addition to pattern similarity that can help characterize early developmental divergence between two species.

Applying TimeMeter to human ES cells and mouse EpiS time series gene expression datasets during neural differentiation, we found that the d*N*/d*S* ratio is significantly lower in STP genes than in genes with dissimilar temporal patterns. The results suggest that STP genes involved in neural differentiation in mice and humans are under strong negative selection during evolution.

We compared mouse limb regeneration with axolotl blastema differentiation, and detected 38 STP genes between these two processes. Among the enriched GO terms, telomere maintenance and organization terms are particular interesting. In vertebrates, regenerative abilities decline with aging (59), and telomere length is known to be associated with aging. Although studies have shown that telomerase is essential for certain tissues (e.g. cardiovascular (54) and kidney (55)) repairing and regeneration, the general role of telomere in regeneration is largely unknown. The enriched telomere related GO terms in STP genes of mouse limb regeneration and axolotl blastema differentiation highlight the potential general role of telomeres in regeneration.

## DATA AVAILABILITY

TimeMeter is available at (http://www.morgridge.net/TimeMeter.html).

The mouse digits regeneration RNA-seq data generated in this study is available at GEO with accession number GSE130438.

## Supplementary Material

gkaa142_Supplemental_Files
