# Supplementary material for: TimeMeter assesses temporal gene expression similarity and identifies differentially progressing genes
Source: Nucleic Acids Res. 2020 Mar 3;48(9):e51. doi: 10.1093/nar/gkaa142 (PMC7229845; doi:10.1093/nar/gkaa142)
Supplement: gkaa142_Supplemental_Files [file gkaa142_supplemental_files.zip › List_of_Supplementary_Data.docx]

# Supplementary Data

**Figure S1**: Illustration of TimeMeter by simulated high density discrete time series data (time shift patterns). (a-d) The query is delayed by 5 days. (e-h) The query is delayed by 15 days. (i-l) The query is delayed by 30 days.

**Figure S2**: Illustration of TimeMeter by simulated high density discrete time series data (different speed in dynamical change patterns). (a-d) The query is 1.2-fold faster in dynamical change than the reference. (e-h) The query is 1.4-fold faster in dynamical change than the reference. (i-l) The query is 1.6-fold faster in dynamical change than the reference.

**Figure S3**: Simulation study of how the data noise and sampling density will affect p-values in TimeMeter. The query and the reference have a different speed in dynamical change patterns (simulated discrete time series data). (a) Increasing the noise level will decrease the power to detect the pattern associations. (b) A higher sampling density will increase the power to detect the pattern associations.

**Figure S4**: Differential progression genes (|PAS|>5) between axolotl and *Xenopus* during early embryo development. (a) Enriched neural development related GO terms in Axolotl advanced genes. (b) Enriched muscle or smooth muscle related GO terms in *Xenopus* advanced genes.

**Figure S5**: Decomposing Barry et al detected STP genes into two lists: “Both” and “Barry et al only”, and performing GO enrichment analysis.

**Figure S6**: Using a subset of Barry et al. time points which can match to known human-mouse Carnegie stage equivalents (*in utero*) to evaluate TimeMeter and Barry et al. methods. (a) Matching Barry et al (*in vitro*) days with embryonic day equivalents (in utero). We added 6.5 days to mouse epiblast stem (EpiS) cells and 15 days to human embryonic stem (ES) cells to transposing Barry et al (in vitro) days to embryonic day equivalents (in utero) (a detailed explanation for this transposition can be found in Barry et al). The perfectly matched (rounded to integer) of human-mouse day pairs are highlighted as blue dots. (b) Cumulative distribution function (CDF) plot of human-mouse temporal gene expression Pearson correlation coefficient of “Both” (red color) and “Barry et al only” (green color) STP genes based on a subset of time points matched with human-mouse in utero day equivalents.

**Figure S7**: PAS distribution (density) of genes with similar temporal patterns (STP) between human ES and mouse EpiS during neural differentiation. The black and red lines indicate before and after adjusting species difference for calculating PAS values.

**Figure S8**: The scenarios (experimental design) for which one can and cannot apply TimeMeter, and alternative solutions. (a-e) Different sampling scenarios (experimental design) that can apply TimeMeter directly. (f) TimeMeter does not allow uneven sampling (large gaps in time). An alternative approach is to transform real time to pseudo-time (time order). In this scenario, TimeMeter will aim to detect “time-order similar genes”.

**Table S1**: Genes with similar temporal similar patterns (STP) between axolotl and *Xenopus* during early embryo development.

**Table S2:** Enriched GO terms (BH adjusted P-value < 0.05) for STP genes between axolotl and *Xenopus* during early embryo development.

**Table S3**: Enriched GO terms (BH adjusted P-value < 0.05) for axolotl advanced (PAS>4) and *Xenopus* advanced (PAS<-4) genes.

**Table S4**: Genes with similar temporal similar patterns (STP) between human embryonic stem (ES) cells (from day 0 to day 42) and mouse epiblast stem (EpiS) cells (from day 0 to day 21) during neural differentiation.

**Table S5**: Genes with similar temporal similar patterns (STP) during mouse limb regeneration and axolotl blastema differentiation.
