# Supplementary material for: TimeMeter assesses temporal gene expression similarity and identifies differentially progressing genes
Source: Nucleic Acids Res. 2020 Mar 3;48(9):e51. doi: 10.1093/nar/gkaa142 (PMC7229845; doi:10.1093/nar/gkaa142)

Supplementary Figure S8

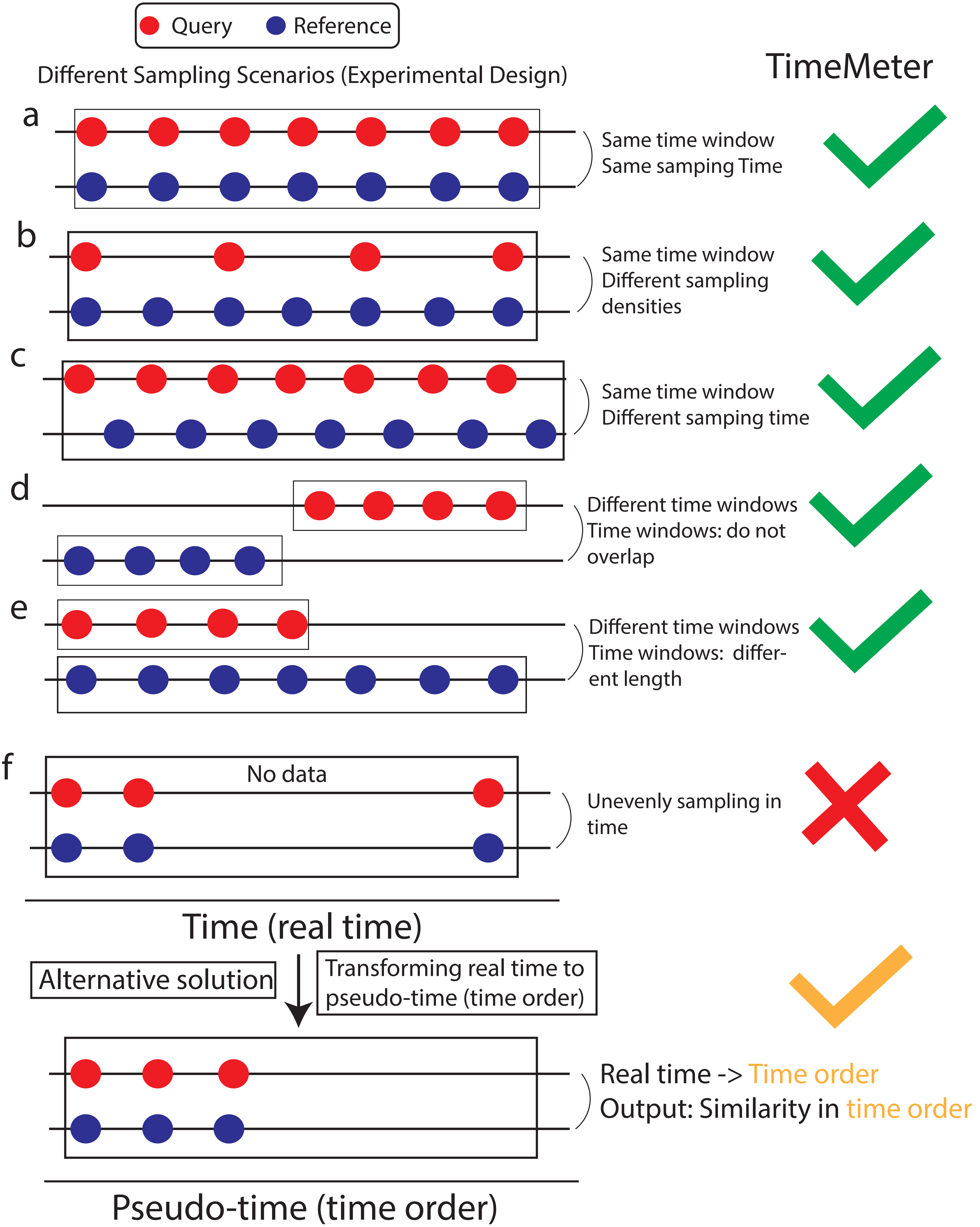

Supplement: gkaa142_Supplemental_Files [file gkaa142_supplemental_files.zip › Supplementary_Fig.S8.TimeMeter_Use_Conditions.pdf]
