# Supplementary figures and images for: TimeMeter assesses temporal gene expression similarity and identifies differentially progressing genes
Source: Nucleic Acids Res. 2020 Mar 3;48(9):e51. doi: 10.1093/nar/gkaa142 (PMC7229845; doi:10.1093/nar/gkaa142)

Supplementary Figure S1

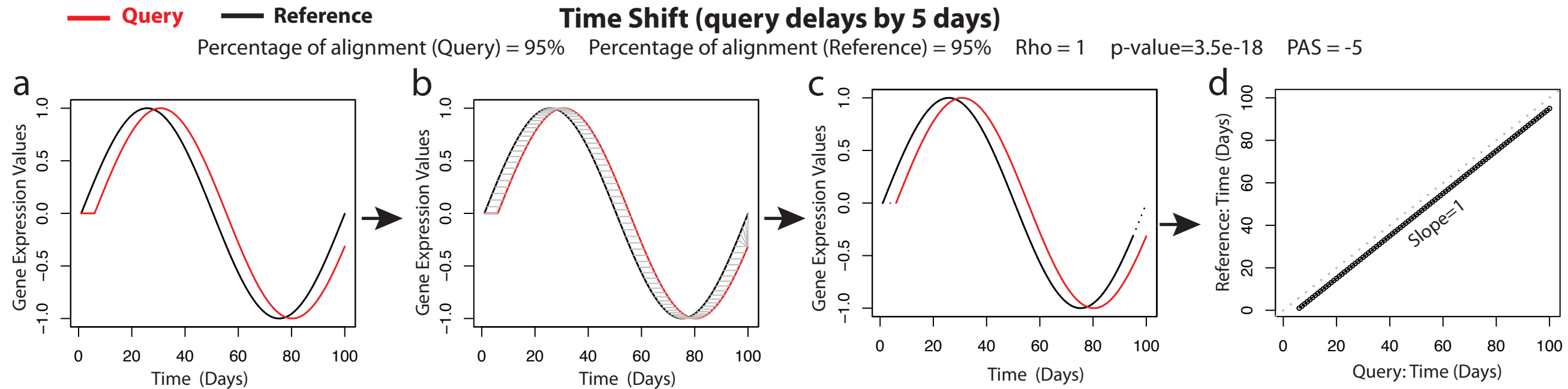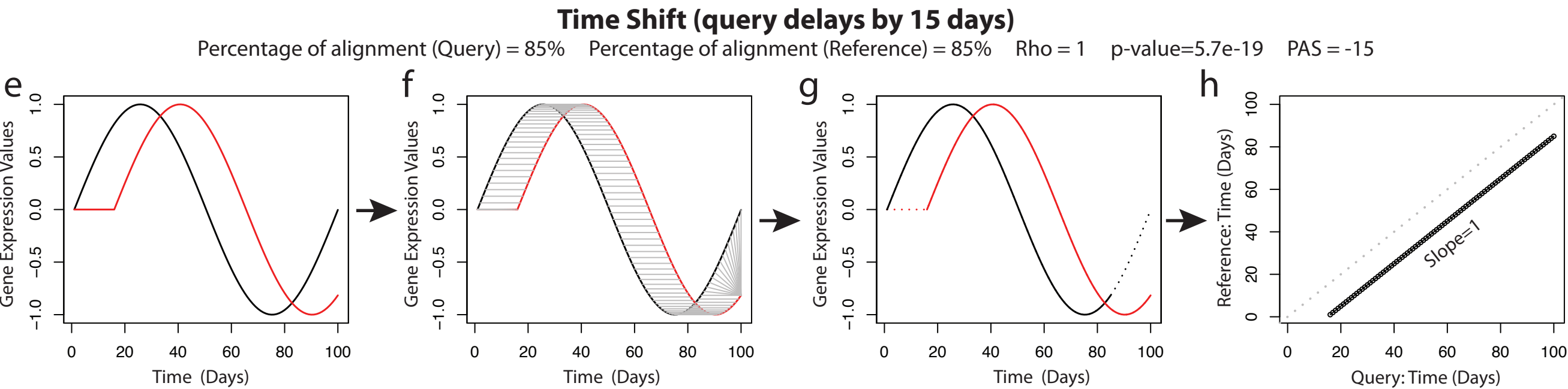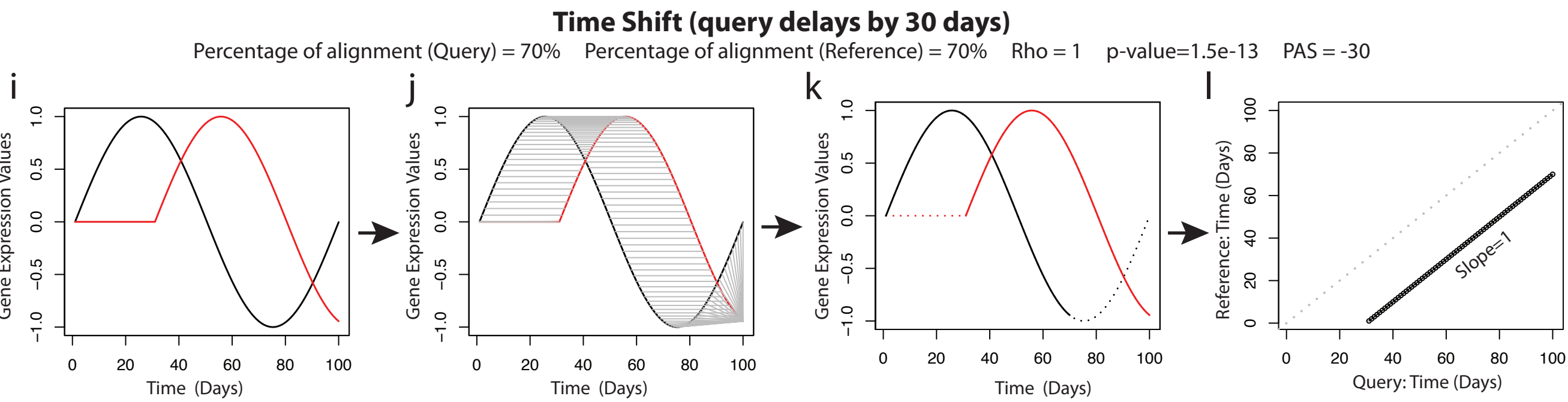

Supplement: gkaa142_Supplemental_Files [file gkaa142_supplemental_files.zip › Supplementary_Fig.S1.timeShift.pdf]

Supplementary Figure S2

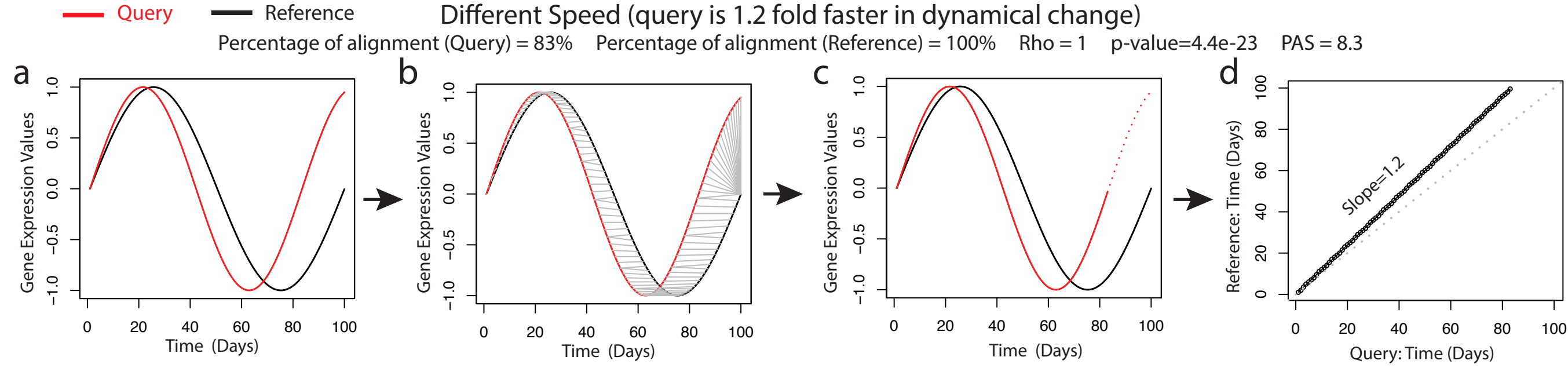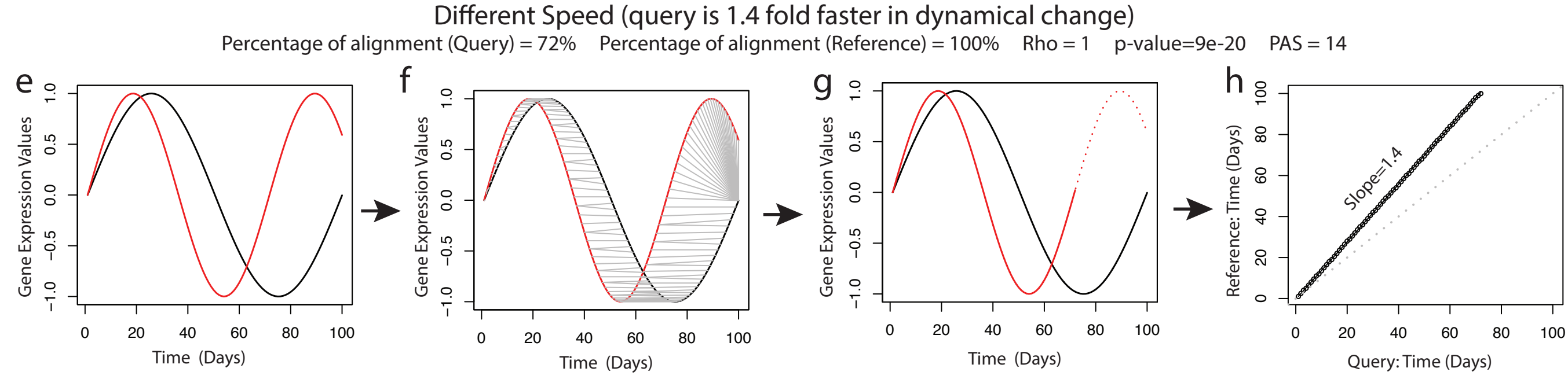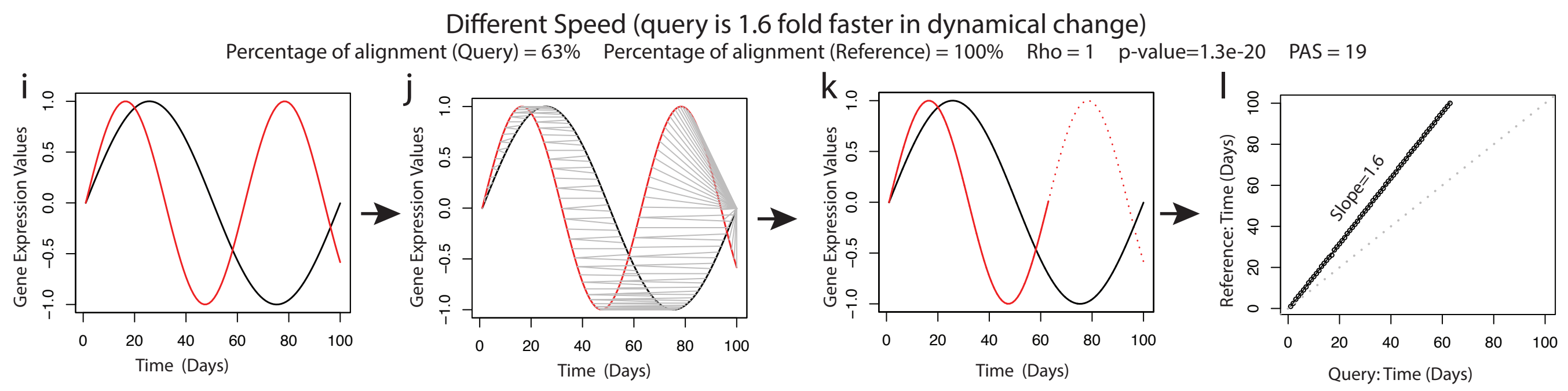

Supplement: gkaa142_Supplemental_Files [file gkaa142_supplemental_files.zip › Supplementary_Fig.S2.DIfferentSpeed.pdf]

Supplementary Figure S3

a

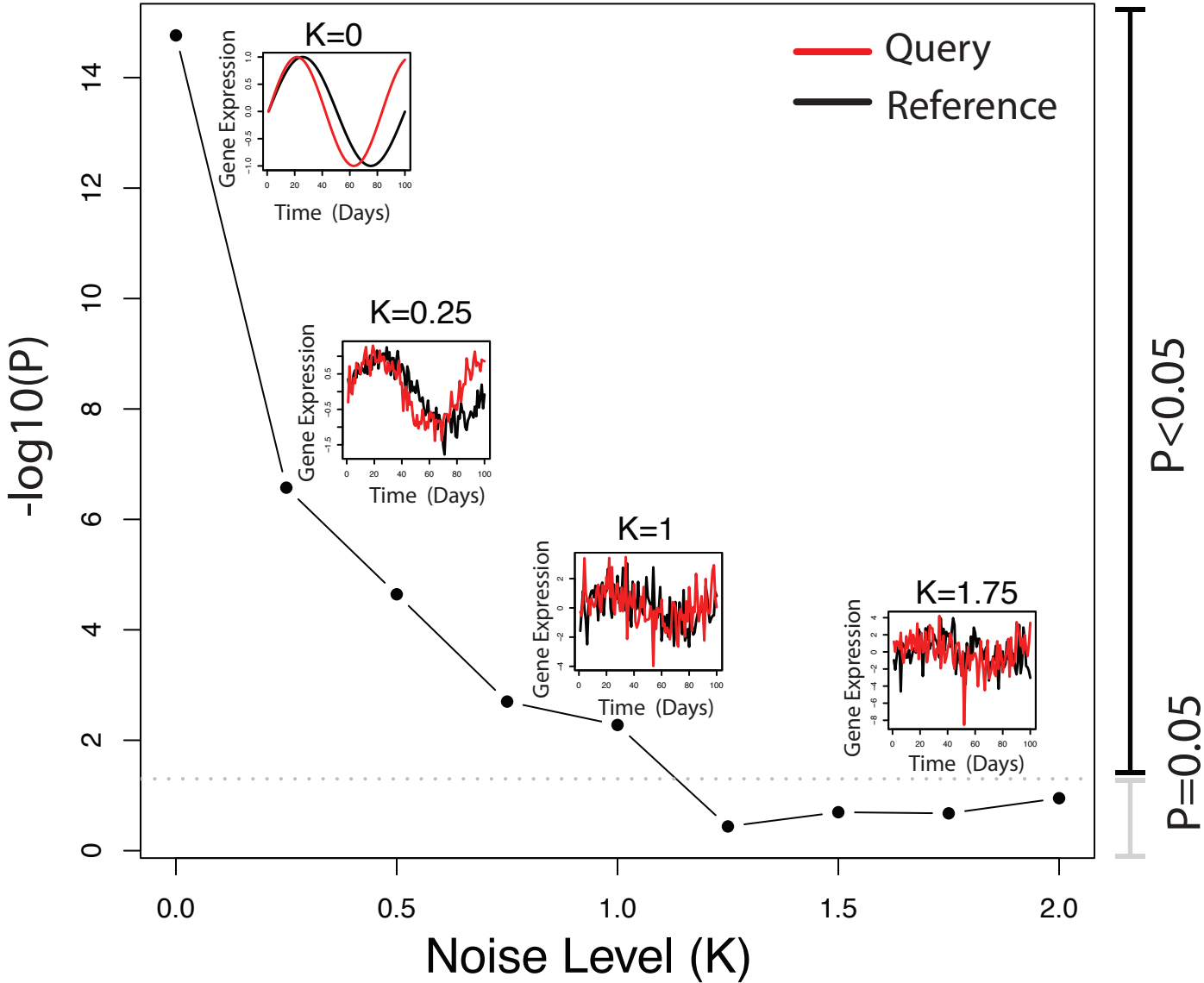

b

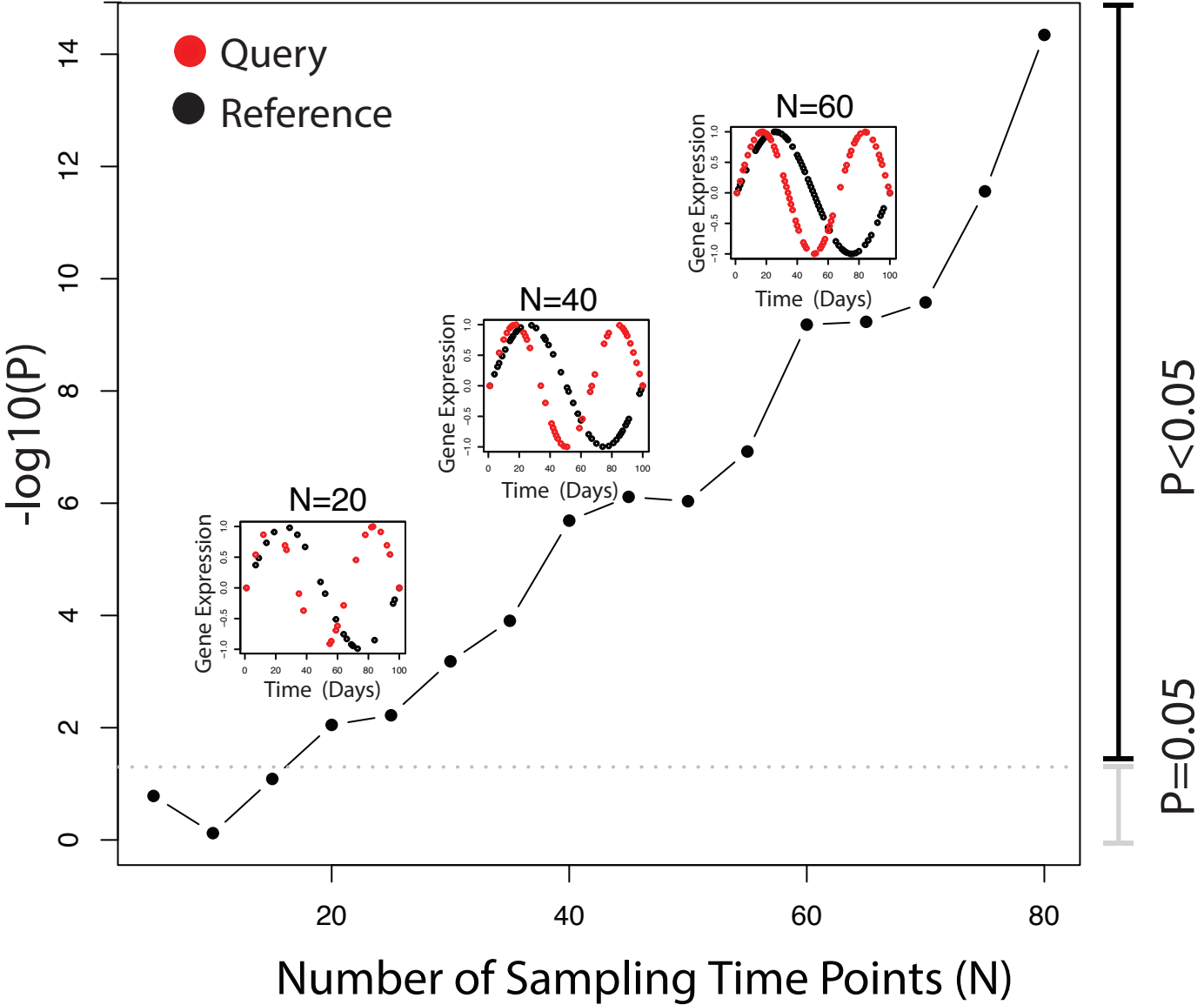

Supplement: gkaa142_Supplemental_Files [file gkaa142_supplemental_files.zip › Supplementary_Fig.S3.P_value_DifferentSpeed.pdf]

Supplementary Figure S5

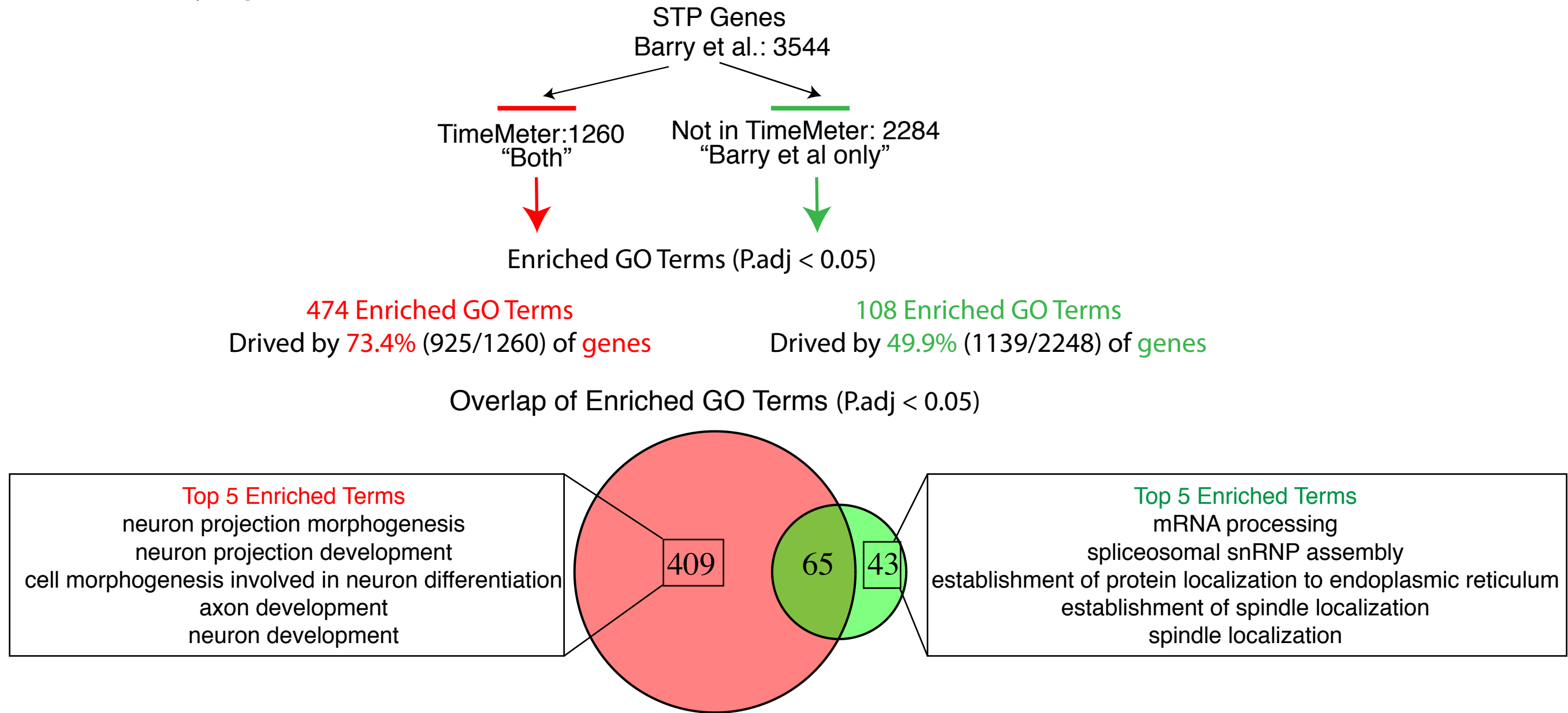

Supplement: gkaa142_Supplemental_Files [file gkaa142_supplemental_files.zip › Supplementary_Fig.S5.All_Enriched_GO_and_driving_genes.pdf]

# Supplementary Figure S7

Density (PAS)

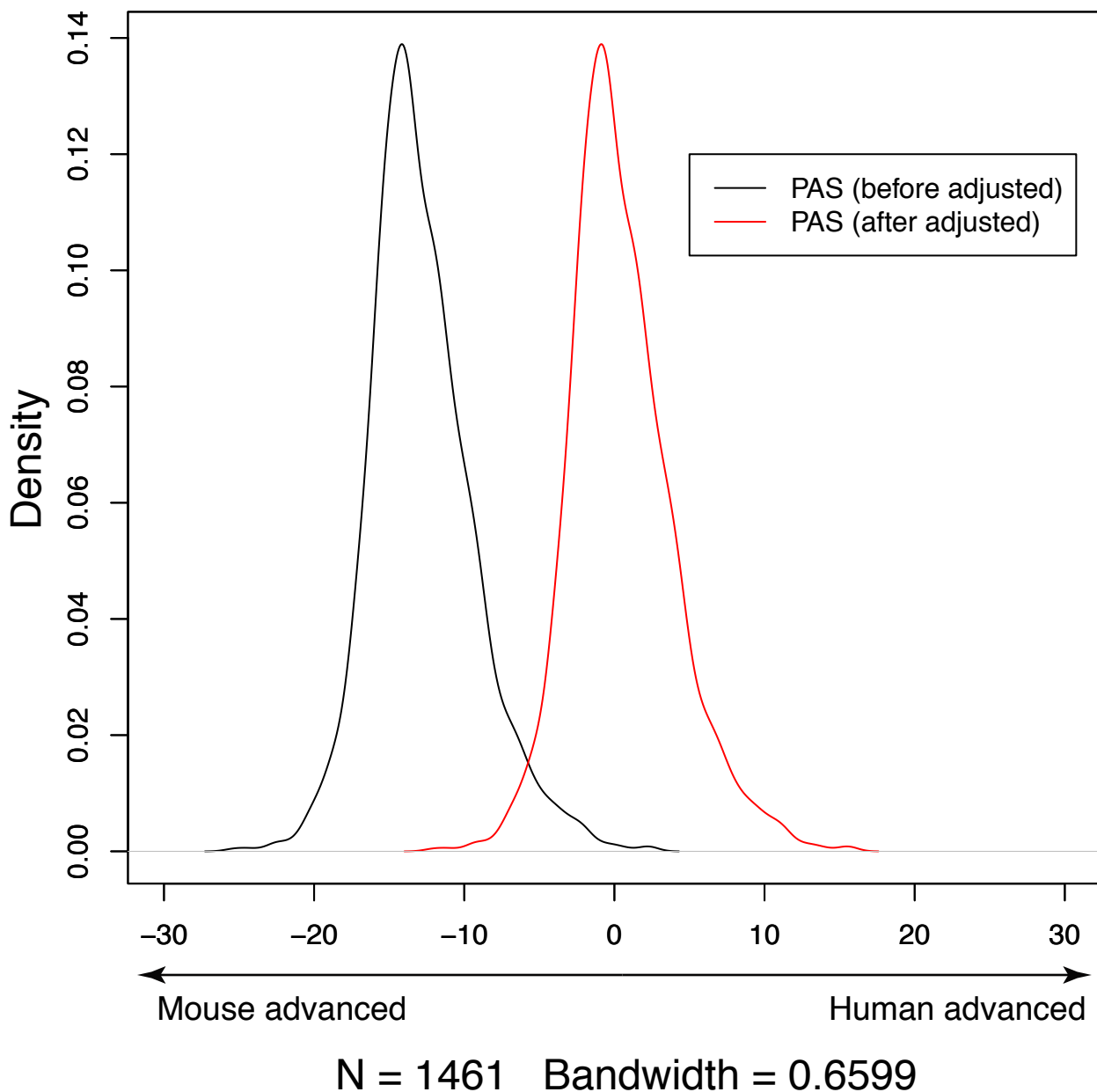

Supplement: gkaa142_Supplemental_Files [file gkaa142_supplemental_files.zip › Supplementary_Fig.S7.density_PAS_before_and_after_adjust.pdf]
